# Potentiality of Using Luojia1-01 Night-Time Light Imagery to Estimate Urban Community Housing Price—A Case Study in Wuhan, China

**DOI:** 10.3390/s19143167

**Published:** 2019-07-18

**Authors:** Chang Li, Linqing Zou, Yijin Wu, Huimin Xu

**Affiliations:** 1 Key Laboratory for Geographical Process Analysis & Simulation, Hubei Province, Central China Normal University, Wuhan 430079, China; 2 College of Urban and Environmental Science, Central China Normal University, Wuhan 430079, China; 3 School of Economics, Wuhan Donghu University, Wuhan 430212, China

**Keywords:** Luojia1-01, night-time light intensity (NTLI), community housing price (CHP), geographically weighted regression (GWR), small-scale, spatial analysis, quantitative modeling, potentiality, coupling mechanism

## Abstract

The first professional night-time light remote sensing satellite in China, Luojia1-01, has raised the resolution of night-time light data to 130 m, which provides a possibility for the study of small-scale night-time light. This paper is the first research on spatial analysis and quantitative modeling between night-time light intensity (NTLI) and community housing price (CHP) on a small scale by using the Luojia1-01 night-time light imagery. This paper takes Wuhan as the research area, CHP data obtained by web-crawler technology as the research object, combines Luojia1-01 data, and carries out spatial correlation analysis and quantitative modeling on a small scale for them. The experimental results show that there is a strong linear positive correlation between the NTLI and CHP based on geographically weighted regression (GWR), and the CHP data in Wuhan have obvious spatial non-stationarity. Moreover, the coupling mechanism between the NTLI and CHP is also revealed. We can conclude that there is potential for estimating the CHP by using Luojia1-01 night-time light imagery.

## 1. Introduction

Urban housing prices have historically been an important part of urban geography, urban sociology, and regional economic research. Since the 21st century, the real estate industry has developed rapidly, and its position in the national economy has become stronger and stronger [1]. Real estate prices are related not only to the production and life of ordinary people but also to the potential of a city’s development and the stability of the whole national economy. Many studies have shown that housing prices are influenced by market demand, supply, capacity, and external factors [2,3,4,5], and the housing prices among different regions are also interdependent. Taking spatial correlation as an influencing factor in housing price research can provide a more effective basis for the spatial analysis of housing prices within a certain region [6].

In recent years, the rise of night-time light remote sensing technology has provided a new way to explore urban and urbanization issues [7,8,9]. Currently, the most widely used night-time light remote sensing images come from Defense Meteorological Satellite Program - Operational Linescan System (DMSP-OLS) and The Suomi National Polar-orbiting Partnership - Visible Infrared Imaging Radiometer (NPP-VIIRS) night-time light satellites in the United States [10,11,12]. Their spatial resolutions are approximately 1000 m and 500 m, respectively. Due to the limitation of the image resolution, the current research on night-time light is mostly on a large scale, while research on small-scale night-time light is scarce. Currently, there is no research on quantitative modeling of night-time light data and housing prices at a small scale in China or abroad. The first professional night-time light remote sensing satellite in China, Luojia1-01, improves the resolution of night-time light data to 130 m, while the area of general residential areas is at least 10,000 m^2^. The improvement of remote sensing image accuracy meets the minimum area requirement of residential areas, which brings new possibilities and opinions for the quantitative modeling of night-time light and housing prices on a small scale.

Tan et al. [13] introduced a time-aware latent hierarchical model to capture underlying spatiotemporal interactions behind the evolution of house prices, and the proposed framework was examined on a large-scale data set of the property transaction in Beijing. Bera et al. [14] discussed the value of local environmental amenities based on the hedonic price model. Locally weighted hedonic regressions were used instead of global hedonic regression. The estimation shows that environmental and land-use attributes have location characteristics, and there is a significant correlation with housing prices. Du et al. [15] simulated the spatial heterogeneity of housing prices and explored the spatial discrepancy of landscape effects on the real estate value in Shenzhen, China. Combining the official housing transaction records and housing attributes from open data on field surveys, this paper explored the spatial change of housing prices by using the geographically weighted regression model (GWR) and analyzed the impact of attributes on housing prices. In summary, there are few reports on the use of comprehensive objective evaluation indicators, especially remote sensing data for spatial analysis and quantitative modeling of housing prices on a small scale.

Night-time light can be linked to human activities in some regions [16,17,18,19,20], and night-time light data have been widely used as an effective measure to monitor urbanization dynamics and socio-economic activities [21,22,23]. Yu et al. [24] used NPP-VIIRS night-time light data and taxi GPS tracking data for estimation of population surface enhancement. Li et al. [25] used Luojia1-01 and VIIRS night-time light images to compare several methods of extracting urban areas. The experimental results show that using night-time light images can greatly improve the accuracy of urban area extraction, while Luojia1-01 data have higher resolution and more abundant spatial information and can obtain better recognition results than previous night-time light data. Zhang et al. [26] compared Luojia1-01 night-time light data with NPP/VIIRS data in the context of simulating socio-economic parameters. The results show that Luojia1-01 night-time light data have a better ability to model socio-economic parameters than NPP/VIIRS data, and the former can be used as an effective tool to build a socio-economic parameters model. There is a significant positive correlation between the two types of night-time light data and 10 socio-economic parameters. Li et al. [27] suggested using night-time light data as an alternative indicator for assessing the comprehensive indicators of regional sustainable development. As a comprehensive indicator, night-time light is more objective than statistical data, more convenient to obtain, and has a certain periodicity. Li et al. [28] evaluated the spatial property and change detection capability of Luojia1-01 through the preliminary investigation of Luojia1-01 night-time light imagery. The research shows that Luojia1-01 images are effective data sources for spatiotemporal distribution of night-time light and its associated socioeconomic attributes.

In this paper, Wuhan city is used as a research area. Community housing price (CHP) data in Wuhan are obtained through web-crawler technology. Combining with Luojia1-01 night-time light imagery, this paper uses the exploratory spatial data analysis (ESDA) to reveal the spatial dependence and heterogeneity through the analysis of spatial autocorrelation, constructs a GWR for analysis at a small scale, analyzes its spatial differentiation characteristics and its variation law, and studies the potentiality of estimating CHP by using Luojia1-01 night-time light imagery. This study has the following innovations and contributions:

(1) The spatial clustering analysis of small-scale housing prices using Luojia1-01 data is first proposed to analyze whether the night-time light intensity (NTLI) and CHP in small-scale (community) areas are spatially identical. Currently, there are few applications of Luojia1-01 data, and no relevant reports on the above studies have been found in previous studies of night-time light. Luojia1-01 data contain more spatial details than traditional night-time light data. Based on the ESDA method, the spatial dependence and heterogeneity of small-scale housing prices are revealed through spatial autocorrelation analysis, which enriches the theory and method of small-scale night-time light remote sensing geoscience application and makes up for the deficiency of macro-analysis.
(2) Under the scale of residential communities, the spatial quantitative relationship between Luojia1-01 NTLI and CHP is explored, and the GWR model is first established for them.
(3) The CHP data used in this paper originate from the manual data mining of the large-scale real estate information network in China. It has the characteristics of strong timeliness, high accuracy and accurate spatial location. However, traditional housing price research mostly uses social and economic statistics, often takes the administrative region as the basic statistical unit, and has some problems such as data lag.
(4) The coupling mechanism between the NTLI and CHP is the first to be revealed and analyzed, which is explored from the point of view of loop, central city and human activities.

## 2. Materials and Methods

### 2.1. Study Area

**Figure 1:**
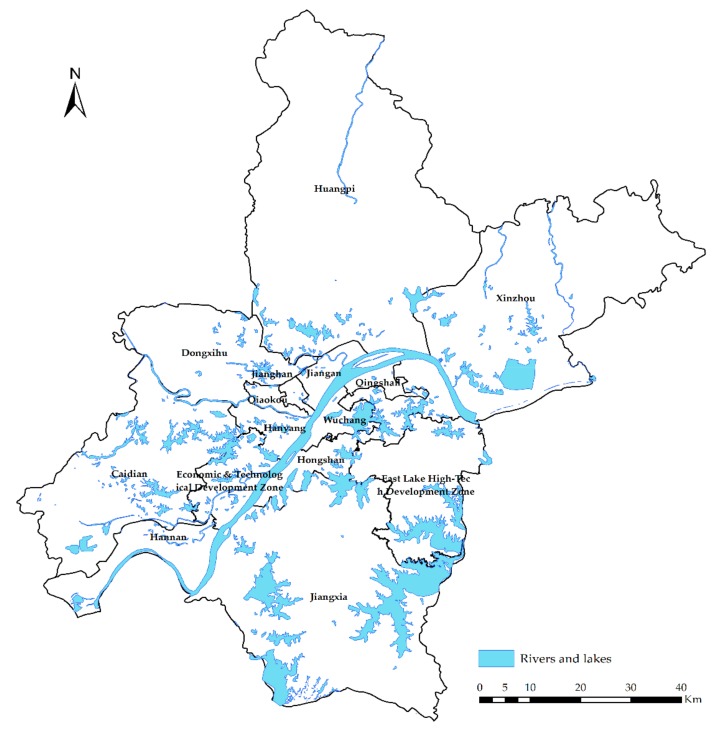
Map of the administrative divisions of Wuhan city.

This paper chooses Wuhan city, Hubei Province, as the research area. Wuhan is the capital of Hubei Province (Figure 1). It is located at 113°41′ E~115°05′ E, 29° 58′ N~31°22′ N and is the central city of central China. Wuhan has a land area of 8569.15 square kilometers, a built-up area of 723.74 square kilometers, and a resident population of 11.081 million. It covers 15 administrative districts, including Jiang’an, Jianghan, Qiaokou, Hanyang, Wuchang, Qingshan, Hongshan, Caidian, Jiangxia, Huangpi, Xinzhou, Dongxihu, Hannan, East Lake High-Tech Development Zone, and the Economic and Technological Development Zone [29].

There are some reasons to choose Wuhan city as the research area.

(1) Location factors. As the central city of central China, Wuhan is the center of China’s economic geography.
(2) The economic development level of Wuhan is very representative. Wuhan’s economic development level is on the medium to high level in the whole country, which can represent most cities with similar levels of economic development.
(3) The Luojia1-01 satellite was developed and produced by Wuhan University. It is of special significance to select Wuhan as the research area for Luojia1-01.

### 2.2. Data

#### 2.2.1. Luojia1-01 Data

**Figure 2:**
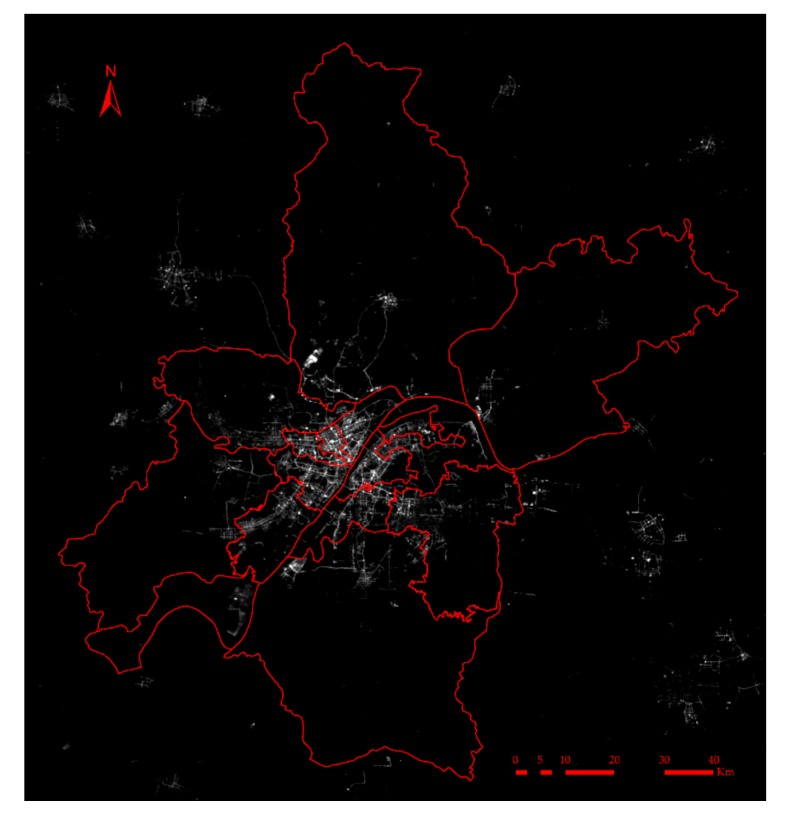
Luojia1-01 night-time light imagery (NTLI) of Wuhan in 2018.

The Luojia1-01 satellite, launched on 2 June 2018, is the world’s first professional night-time light remote sensing satellite. It has an imaging capability with 130 m resolution and a 250 km-wide field. It provides an objective basis for the study of socio-economic parameter estimation and ecological environmental disaster monitoring. These data can be downloaded free of charge from the High Resolution Earth Observation System of Hubei Data and Application Center [30]. Since the radiation calibration of the Luojia1-01 image is still being improved, digital values (DN) are used for analysis in this experiment. In this paper, 11 night-time light images covering the whole city of Wuhan are selected from the Luojia1-01 satellite images (Figure 2). The acquisition time of the images includes June, September, and October.

#### 2.2.2. CHP Data

(1) CHP data used in this paper are derived from the manual data mining of the second-hand housing sales platforms in small- and medium-sized districts of the large-scale real estate information network “Lianjia” website [31] and “Anjuke” website [32]. The second-hand housing sales data platform of Lianjia and Anjuke are one of the most important real estate information platforms in China. These data cover a wide range of areas and have high timeliness.

First, web-crawler technology is used to crawl the name of the community in the real estate information network, the unit price of all the second-hand houses in the community, and the latitude and longitude information of the community. The average value of the unit price of the second-hand houses sold in the same month in each community is taken as the average housing price of the community. For the same community, if there are differences in housing prices between the Lianjia and Anjuke platforms, the average is taken; if only one platform has data, the data of that platform is taken as the criterion. These housing price data mainly focus on the listing price of the third-class real estate market (second-hand housing transactions), which can more realistically reflect the actual situation of housing prices in this community.

**Figure 3:**
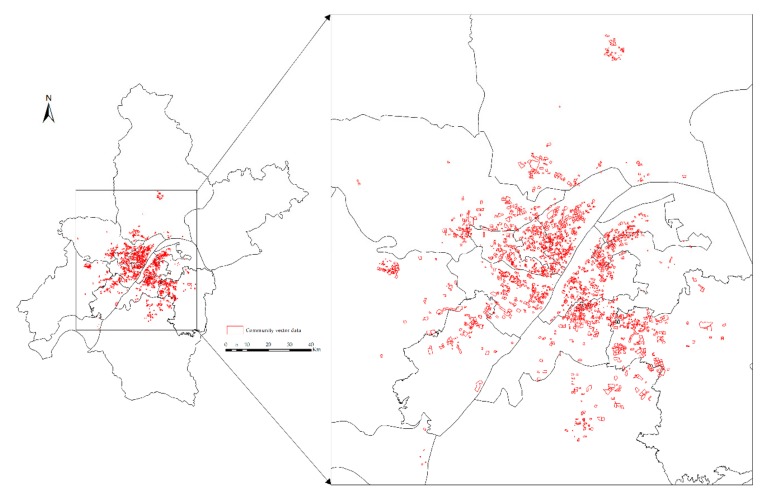
Community vector data set.

(2) The community vector data set includes 2349 data points from 15 districts (Figure 3). The vector data set contains location information, community name and its boundary. For crawler data and community vector data, community name and location are their common attributes.

### 2.3. Method

**Figure 4:**
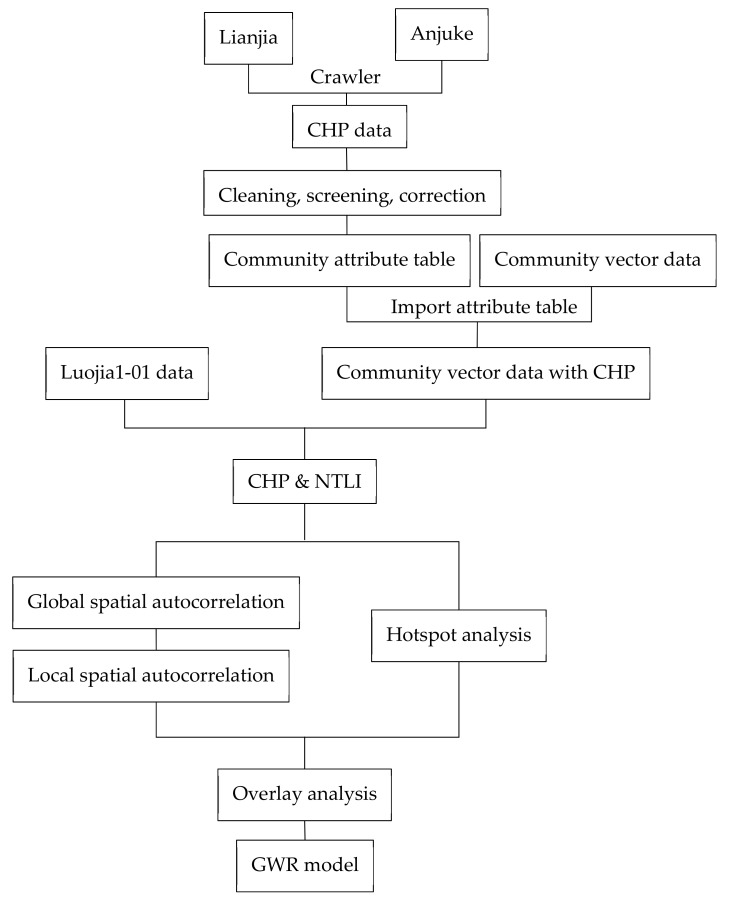
Technology roadmap.

The technical roadmap for this article is shown in Figure 4.

(1) First, web-crawler technology is used on the real estate information network to crawl the property data of housing prices in Wuhan communities. The crawled data are cleaned to obtain the community attribute table. The community attribute table is imported into the community vector data, and the community vector data set with crawled data is obtained.
(2) The community vector data are superimposed with Luojia1-01 images to obtain the NTLI of each community. For each community, the average value of 11 night-time night images is taken as their NTLI.
(3) The method of global spatial autocorrelation is used to explore the spatial distribution and aggregation degree of the NTLI and CHP, respectively. The method of local autocorrelation is used to further judge the aggregation state of the local areas and measure the local spatial correlation and spatial difference between each area and adjacent areas.
(4) Hotspot analysis of the CHP and NTLI is carried out. Overlay analysis is carried out to identify the common clustering space of the two.
(5) GWR is used to address spatial non-stationarity and to analyze the spatial distribution of housing prices.

#### 2.3.1. Data Pre-Processing

(1) Luojia1-01 Data Processing: Since the radiometric calibration of Luojia1-01 imagery is still under improvement, the numerical value (DN) is used for analysis in this study [25].
(2) CHP data processing: By means of web-crawler technology, information such as community name, longitude and latitude, housing price, and the area to which they belong is crawled on the Lianjia website and the Anjuke website. The average unit price of second-hand houses sold in each district in the same month is taken as the average house price of the community. For the same community, if there are differences in housing prices between the Lianjia and Anjuke platforms, the average is taken; if only one platform has data, the data of that platform is taken as the criterion.
(3) After crawled housing price data are acquired, these data are cleaned and corrected, including collinear data processing, merging, and repetitive deletion, to remove outliers.
(4) Through the same field (community name) in the housing price data and the community vector data set, the crawled CHP data are added to the attribute table of the community vector data set. The community vector data set with the crawled CHP is obtained.
(5) The community vector data are overlaid with night-time light images to obtain the night-time light values of the different communities. For each community, the average value of the 11 night-time light images is taken as their NTLI.

#### 2.3.2. Spatial Clustering Analysis

ESDA is one of the most commonly used methods in spatial clustering analysis. ESDA aims to detect spatial attributes of data and reveal spatial dependence and heterogeneity through spatial autocorrelation analysis. There are two kinds of spatial data exploratory analysis methods: Global statistics, which mainly explores the distribution characteristics of an attribute in a region, and local statistics. Through the analysis of information in sub-regions, we can determine whether there is heterogeneity in regional information.

(1) Global spatial autocorrelation analysis

Global spatial autocorrelation analysis can measure the degree of spatial correlation and spatial difference among regions as a whole. *Global Moran’s I* statistic is a commonly used measure of global spatial autocorrelation. Its expression is as follows [33]:

(1) $$I=\frac{N}{\sum_{i=1}^{N}\sum_{j=1}^{N}W(i,j)}\frac{\sum_{i=1}^{N}\sum_{j=1}^{N}W(i,j)(X_{i}-\bar{X})(X_{j}-\bar{X})}{\sum_{i=1}^{N}{\left(X_{i}-\bar{X}\right)}_{i}^{2}}$$

where *I* is the global spatial autocorrelation index, *N* is the number of communities, and *X_i_* is the observation value of community *i*. In the experiment, the NTLI and CHP are used as the observation values to calculate the global spatial autocorrelation index. $\bar{X}$ is the mean value of *X_i_* and *W*(*i*, *j*) is the spatial weight matrix between communities *i* and *j*, which can be constructed based on the adjacency criterion or distance criterion [34]. Among them, W(i,i)=0.

The spatial connection matrix is generally expressed as an N-dimensional matrix W(n×n). In this study, a weight matrix is constructed based on the spatial distance criterion. For the distance matrix, an appropriate threshold *D* is selected and determined by default value of ArcGIS. The weight matrix is defined as follows [34]:

(2) $$W_{d}(i,j)=\{\begin{array}{cc} 1 & d_{ij}\leq D\\ 0 & d_{ij}\geq D \end{array}$$

The value range of *I* is between [-1, 1]. If *I* < 0, it means a negative spatial correlation; if *I* > 0, it means a positive spatial correlation; if *I* = 0, it means that there is no spatial correlation. *Moran’s I* results were tested by the *Z test* [33]:

(3) $$z\left(I\right)=(I-E(I))/\sqrt{\mathrm{var}(I)}$$

Among them, *E*(*I*) is the expectation of the autocorrelation of observation variables, and var(*I*) represents the variance deviation. If |*z*| is greater than 2.58, the significance level is less than 1%, which means that the observed values are very significantly clustered in space; if |*z*| is greater than 1.96, the significance level is less than 5%, which means that the observed values are significantly clustered in space; if *|z|* is less than 1.96, the significance level is more than 5%, which means that the observed values are randomly distributed in space.

(2) Local spatial autocorrelation analysis

Global autocorrelation analysis measures the dependence of the NTLI on the spatial location of housing prices in Wuhan as a whole, but it cannot identify the specific spatial location of clustering or outliers. Therefore, *Local Moran’s I* is used for further analysis between the NTLI and CHP to reflect the similarity and difference of the distribution of the NTLI and CHP.

In essence, *Local Moran’s I* decomposes *Moran’s I* into regional units. Anselin called it local indicators of spatial association (LISA) [35]. Its expression is [35]:

(4) $$\begin{array}{cc} I_{i}=\frac{X_{i}-\bar{X}}{S_{i}^{2}}\sum_{j=1,j\neq i}^{N}W(i,j)(X_{j}-\bar{X}), & S_{i}^{2}=\sum_{j=1,j\neq i}^{N}{\left(X_{j}-\bar{X}\right)}^{2}/(N-1) \end{array}$$

In the formula, the meanings of *N*, *X_i_*, $\bar{X}$, *W* (*i*, *j*) are the same as those in Equation (1). The *Z test* of LISA is the same as Equation (2).

(3) Hotspot analysis

Hotspot analysis can accurately focus on the center and locate the “hot spot” and “cold spot” areas. Hotspot detection is performed by the local Getis-Ord $G_{i}^{*}$ statistic, and then its local autocorrelation is analyzed. The $G_{i}^{*}$ index can well reflect hot and cold spots in the local space. In the experiment, the hotspot analysis is used to detect hot and cold spots of the NTLI and CHP as observation values. The calculation formula can be expressed as follows [35]:

(5) $$G_{i}^{*}=\left(\sum_{j=1}^{n}W_{ij}x_{j}-\bar{X}\sum_{j=1}^{n}W_{ij}\right)/s\sqrt{(n\sum_{j=1}^{n}W_{ij}{}^{2}-{\left(\sum_{j=1}^{n}W_{ij}\right)}^{2})/(n-1)}$$

where *x_j_* is the attribute value of community *j*, which is the NTLI and CHP, *W_ij_* is the spatial weight between community *i* and *j* (this study builds the weight matrix based on the distance criterion) and the same as *W*(*i*, *j*), *n* is the total number of communities, *S* is the standard deviation of the NTLI or CHP in Wuhan, $\bar{X}$ is the average value of the NTLI or CHP and $G_{i}^{*}$ itself is a test statistic that identifies the hot and cold spots of statistical significance. Generally, standardized *Z* values are used to test the significance of the Getis-Ord $G_{i}^{*}$ statistics. The $G_{i}^{*}$ statistics are *Z*-scores, so no further calculations are needed. When the *Z* value is positive and greater than 1.96, it indicates that the night-time light value or housing price around community *i* is higher than the weighted mean value in the threshold range. It belongs to the high-value spatial agglomeration area, which is called the “hot spot” area. Conversely, it is a “cold spot” area. Near zero *Z*-score means that there is no spatial clustering. The greater the absolute value of the $G_{i}^{*}$ index, the higher the degree of aggregation in the study area.

(4) Overlay analysis

The hotspot analysis results of the CHP and NTLI are superimposed. The common hot and cold spot areas are extracted for the next quantitative analysis of the GWR.

#### 2.3.3. Spatial Quantitative Analysis

Since urban housing prices are closely related to spatial location and have spatial autocorrelation characteristics, the GWR model is often used to quantitatively analyze the spatial distribution of the various influencing factors of housing prices.

The GWR model is a spatial analysis model proposed by Fotheringham et al. [36], which can quantitatively describe the relationship between multiple spatial variables. The model considers spatial factors and effects on the basis of the general linear regression model [37,38,39,40,41]. It is a local spatial regression method used to address spatial heterogeneity. The general form of the GWR model is as follows [36,38,42]:

(6) $$y_{i}=\beta_{0}(u_{i},v_{i})+\sum_{k=1}^{p}\beta_{k}(u_{i},v_{i})x_{ik}+\varepsilon_{i},i=1,2,\ldots ,n$$

where $y_{i}$ is the observation value, $\left(u_{i},v_{i}\right)$ is the geographical location coordinate of community *i*, $\beta_{0}\left(u_{i},v_{i}\right)$ is the regression constant of *i*, $\beta_{k}\left(u_{i},v_{i}\right)$ is the *k* regression parameter of community *i*, which is the function of geographical location, *p* is the number of communities, $x_{ik}$ is the value of independent variable $x_{k}$ at *i*, and $\varepsilon_{i}$ is the random error.

In the above model, an observational value is weighted by the nearest neighbor to position *i*. Therefore, the weighting of an observational value does not remain constant during the calculation process but changes with *i*. The estimation of $\beta_{k}\left(u_{i},v_{i}\right)$ can be expressed as [42]:

(7) $$\hat{\beta }(u_{i},v_{i})={\left(X^{T}W(u_{i},v_{i})X\right)}^{-1}X^{T}W(u_{i},v_{i})Y$$

where **X** is an independent variable matrix and is related to NTLI, **X**^T^ is its transpose matrix, ***Y*** is a dependent variable vector and is related to CHP.

In practice, the weight function is usually determined by the Gauss function, which is expressed as follows [41]:

(8) $$W_{ij}=\exp\left(-d_{ij}^{2}/b^{2}\right)$$

where *b* is the bandwidth. The establishment of the bandwidth is very important for the GWR model. The size of the bandwidth directly affects the spatial change of the GWR model. If the data of community *i* are observed, the weights of other points will decrease with increasing distance $d_{ij}$ according to the Gauss curve.

To obtain the best bandwidth, Brunsdon and Fotheringham et al. [43] further applied the Akaike information criterion (AIC) to weight function bandwidth selection in the GWR analysis according to the results of Hurvich et al. [44]. When the AIC of the GWR model is the smallest and the best bandwidth *b* is acquired, its formula is:

(9) $$AIC_{c}=2n\ln(\hat{\sigma })+n\ln(2\pi )+n[(n+tr(S))/(n-2-tr(S))]$$

(10) $$S=X{\left(X^{T}WX\right)}^{-1}X^{T}W$$

where **S** is the hat matrix. Subscript *c* is the estimated value of the revised AIC, *n* is the number of communities, $\hat{\sigma }$ is the standard deviation of the error estimation, *tr*(*S*) is the trace of the *S* matrix of the GWR and a function of bandwidth.

In spatial analysis, observation data are generally sampled according to a given geographical location as a sampling unit. With the change in geographical location, the relationship or structure of the variables will change, which is called “spatial non-stationarity” in geographic information systems (GIS). This spatial non-stationarity generally exists in spatial data. It is difficult to obtain satisfactory results if the traditional linear regression model is used to analyze spatial data. However, the GWR model is used to address this property of spatial data itself by establishing a local regression equation at each point in the spatial range. It explores the spatial variation relationship between variables and its future results. There are obvious advantages in the process of forecasting.

## 3. Results and Discussion

The experimental design of this paper is as follows:

(1) In spatial clustering analysis, the spatial autocorrelation index is used to study the correlation between the attributes of Wuhan community (i.e., CHP and NTLI) in its spatial location and to test whether the attributes of the residential district are significantly correlated with the attributes of neighboring spatial points. If the correlation is positive, it shows that the change trend of the attribute value of a community is the same as that of its adjacent spatial units, while the negative correlation is the opposite.
(2) In spatial quantitative analysis, the specific idea of using the GWR to quantitatively study the spatial heterogeneity of the CHP and NTLI is as follows: First, the spatial structure characteristics of the CHP data are tested. Second, the Gauss function is selected as the weight function, and the AIC method is selected to determine the optimal bandwidth. Then, the GWR model of the CHP in Wuhan is constructed to simulate. Based on this, the model results are tested: Whether the NTLI significantly indicates spatial non-stationarity. Finally, the simulation and test results are analyzed, discussed, and summarized.

### 3.1. Spatial Clustering Analysis

**Figure 5:**
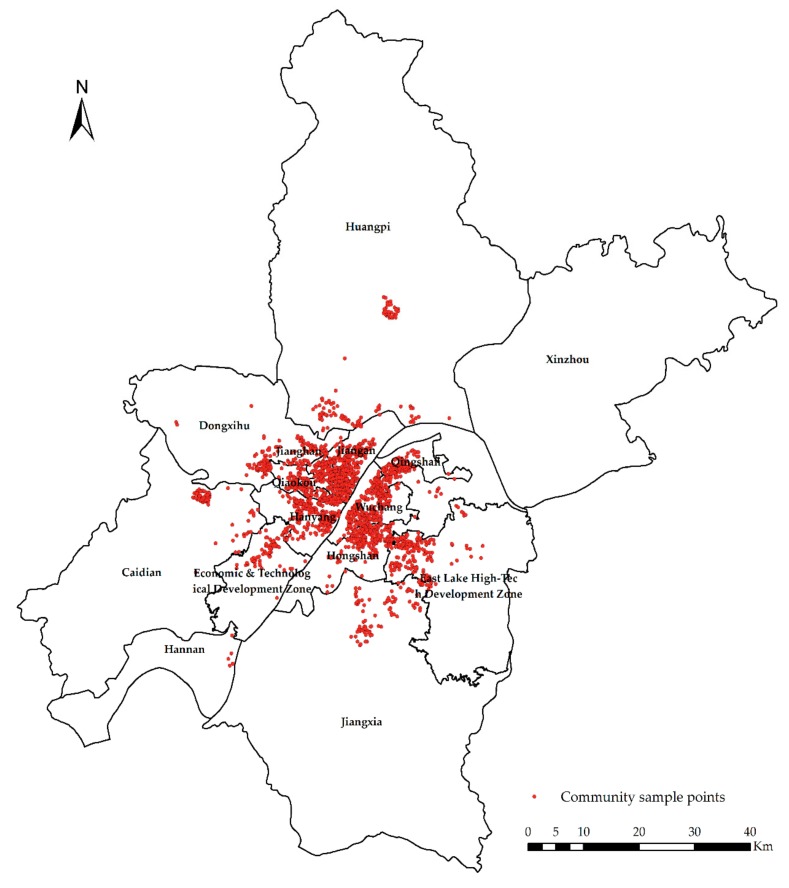
Sample point distribution.

The sample point distribution of this paper is shown in Figure 5.

The *Global Moran’s I* is used to measure spatial autocorrelation for the CHP and NTLI in Wuhan. INVERSE_DISTANCE is used in spatial relations because the adjacent elements have a greater impact on the calculation of target elements than the distant ones. EUCLIDEAN_DISTANCE, the straight-line distance between two points, is used to calculate the distance between each element and its adjacent elements.

The *Global Moran’s I* statistics of the NTLI and CHP in Wuhan are calculated. The results are shown in Table 1.

The calculation results in Table 1 show that both *Moran’s I* index of the CHP and NTLI are greater than 0, both *Z* scores are greater than 2.58 and both *p* values are less than 0.01. Thus, the following results can be obtained: 1) Moran’s I index of both the CHP and NTLI are positive, indicating that both are positively correlated in space, and that the value of the data set used for analysis is proportional to the degree of spatial aggregation. 2) Both *p* values are less than 0.01, so the probability of random generation of both data is only 1% (99% confidence level). 3) Both *Z* scores are more than 2.58, which indicates that both data present obvious clustering characteristics. In general, the *p* value represents the reliability of the data source, and the *Z* score and *Moran’s I* index indicate that the data have obvious regularity.

The clustering degree of high or low values can be measured by the Getis-Ord General G statistics. The results are shown in Table 2.

**Figure 6:**
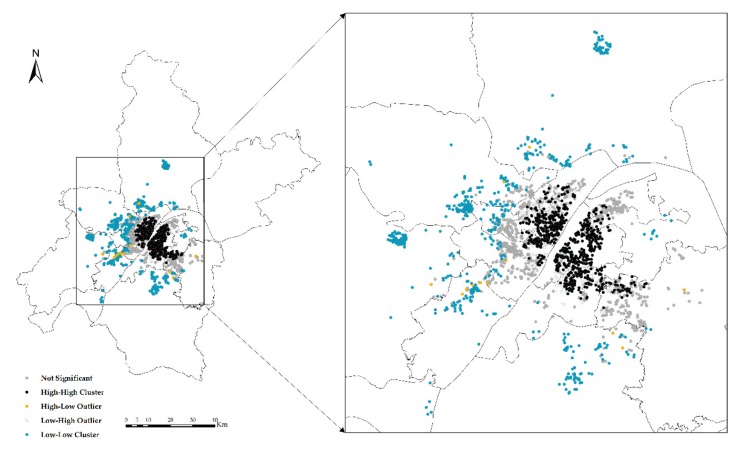
Clustering and outlier analysis of the community housing price (CHP).

**Figure 7:**
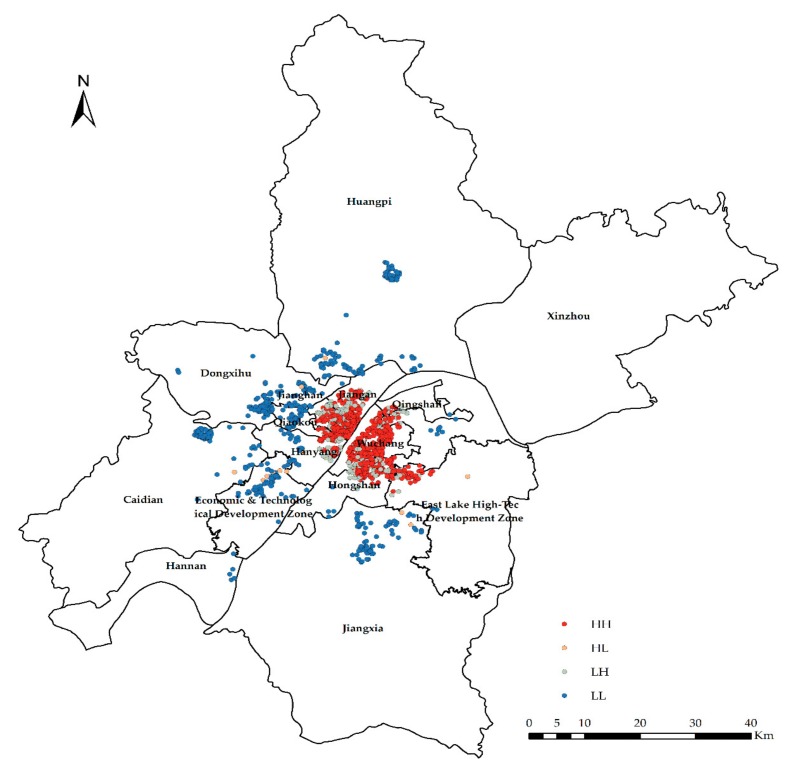
Clustering and outlier type of the CHP.

**Figure 8:**
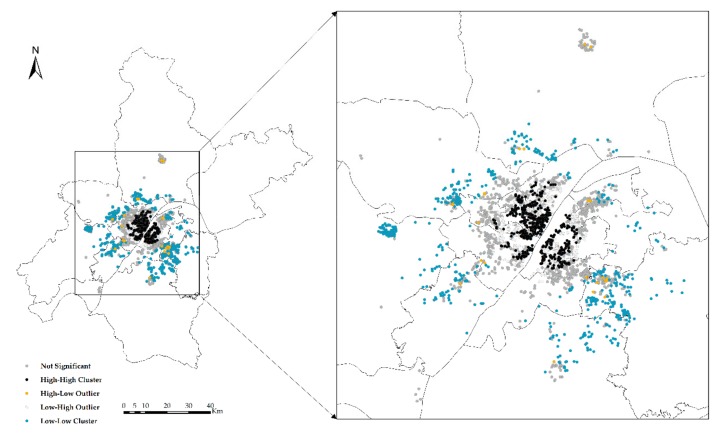
Clustering and outlier analysis of the NTLI.

**Figure 9:**
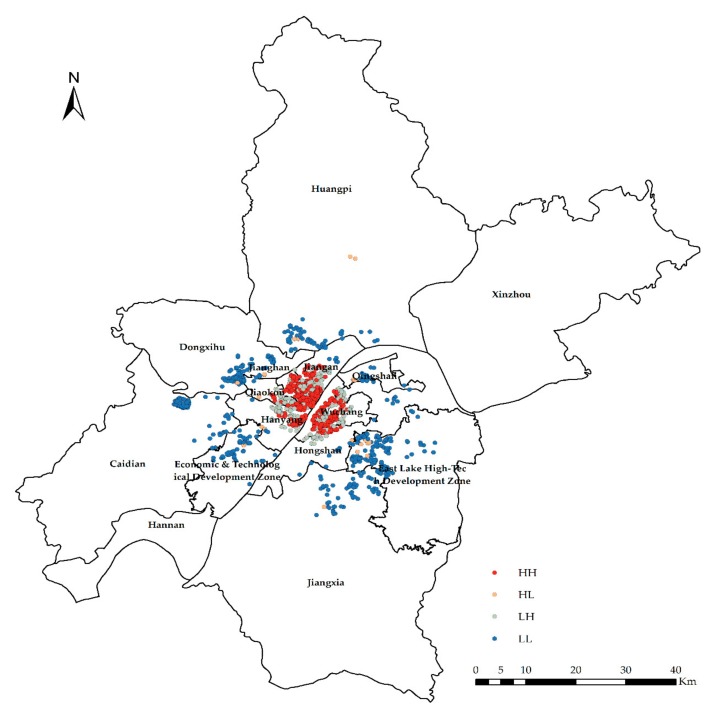
Clustering and outlier type of the NTLI.

The global exponent only tells us whether there are agglomerations or outliers in space, but it does not tell us where. In other words, *Global Moran’s I* only answers yes or no; if global autocorrelation occurs, then local autocorrelation occurs; *Local Moran’s I* tells us where outliers occur or where agglomerations occur, which is a tool to answer where. In the experiment, *Anselin Local Moran’s I* is used to analyze clustering and outliers (Figure 6, Figure 7, Figure 8 and Figure 9).

High-high cluster (HH) indicates that the area with high CHP (or NTLI) is surrounded by other areas with high CHP (or NTLI) or that the level of the CHP (or NTLI) in this area is higher, and the spatial difference of the CHP (or NTLI) in this area is smaller.

Low-high outlier (LH) means that the area with low CHP (or NTLI) is surrounded by other areas with high CHP (or NTLI). In other words, the level of the CHP (or NTLI) in this area is low, and the spatial difference of the CHP (or NTLI) in this area is large.

High-low outlier (HL) means that the area with high CHP (or NTLI) is surrounded by other areas with low CHP (or NTLI) or that the level of the CHP (or NTLI) in this area is high, and the spatial difference of the CHP (or NTLI) in this area is large.

Low-low cluster (LL) indicates that the area with low CHP (or NTLI) is surrounded by other areas with low CHP (or NTLI). In other words, the level of the CHP (or NTLI) in this area is low, and the spatial difference of the CHP (or NTLI) in this area is small.

According to the results of the clustering and outlier analysis of the CHP and NTLI, both their HH clusters occur in the central area, while the LL cluster occurs in the periphery of the city.

In the hotspot analysis, FIXED_DISTANCE_BAND is selected to analyze each element in the adjacent factor environment. The adjacent elements within the specified critical distance will assign a weight of 1, which will have a significant impact on the calculation of the target elements. The adjacent elements outside the specified critical distance will assign weights of zero and will not have any effect on the calculation of the target elements. EUCLIDEAN_DISTANCE is used to calculate the distance between each element and its adjacent elements.

**Figure 10:**
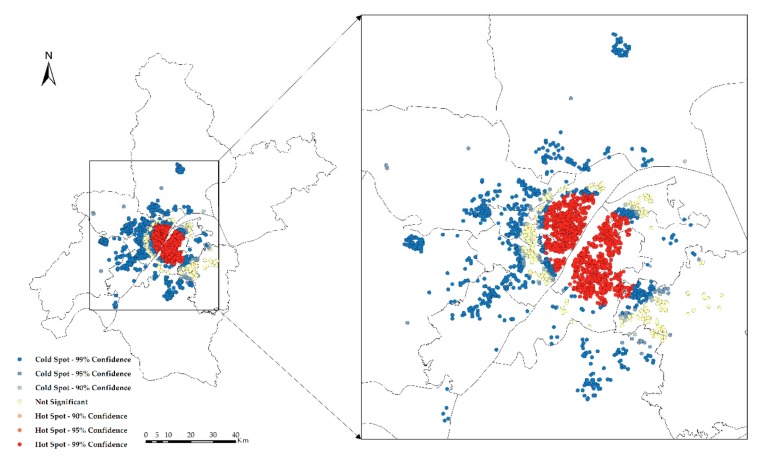
Hotspot analysis of the CHP.

**Figure 11:**
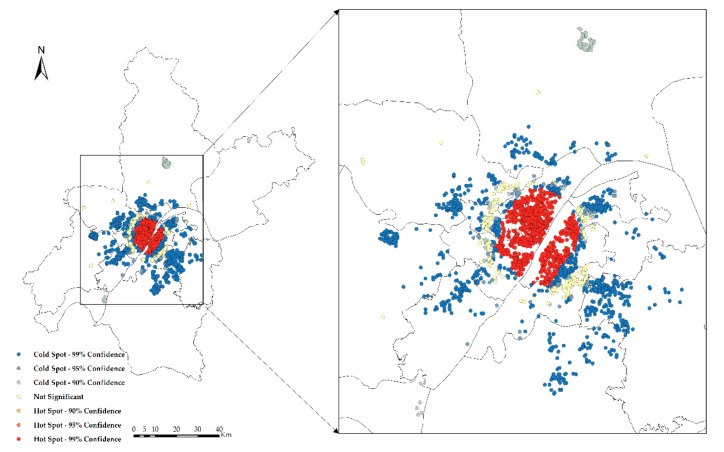
Hotspot analysis of the NTLI.

Hotspot analysis of the NTLI and CHP in Wuhan is carried out, and the results are shown in Figure 10 and Figure 11. From the hotspot analysis, it can be seen that the hot spots of both the NTLI and CHP are concentrated in the city center, the cold spots are concentrated in the periphery of the city, and the transitional zone between the city center and the suburbs is not significant.

**Figure 12:**
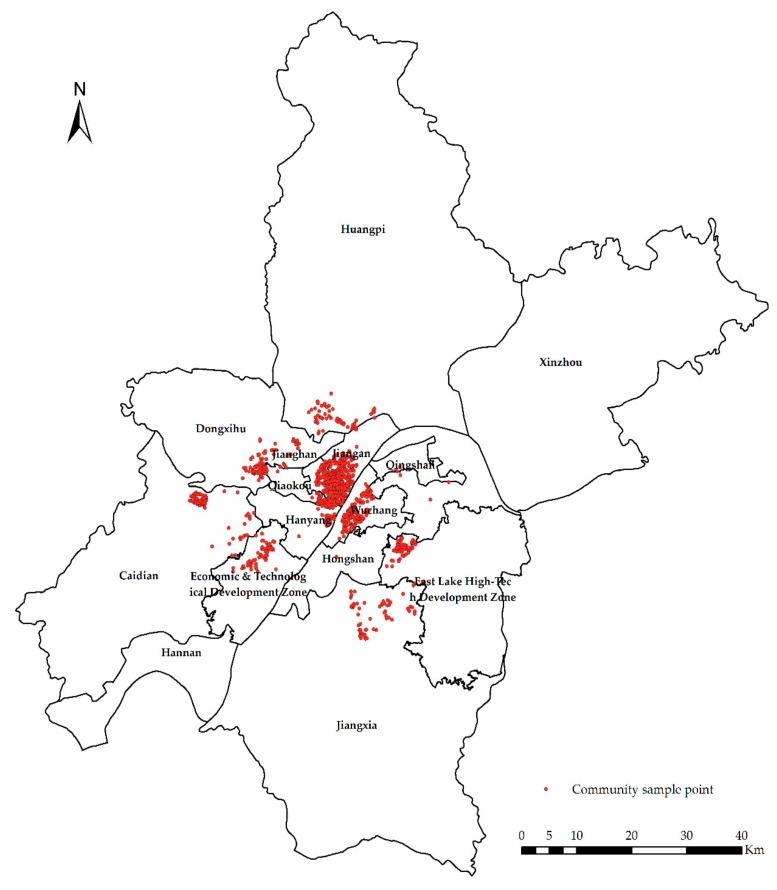
Results of the overlay analysis.

Overlay analyses of the NTLI and CHP in Wuhan are carried out, and the results are shown in Figure 12. From the overlay analysis, it can be seen that the common cold spots and hot spots of the NTLI and CHP are concentrated in the urban center and periphery.

The results of the spatial clustering analysis show the following:

(1) There is a significant positive spatial autocorrelation in the CHP in Wuhan.
(2) There is a significant positive spatial autocorrelation of the NTLI in Wuhan communities.
(3) There is a strong linear positive correlation between the CHP and NTLI in Wuhan.
(4) The HH cluster of both the CHP and NTLI occurs in the central area, and both their LL clusters occur in the periphery of the city.

The CHP in the study area basically conforms to the normal distribution and generally presents the pattern of spatial agglomeration. There is spatial autocorrelation between the CHP and NTLI in Wuhan, which passes the 1% significance test. The CHP in Wuhan decreases from the city center along the traffic ring to the suburbs, forming a circle-like spatial pattern. The NTLI also shows a pattern of spatial agglomeration.

### 3.2. Spatial Quantitative Analysis

The results of GWR and OLS are shown in Table 3.

For the GWR method, its R^2^ and R^2^ adjusted values are above 0.518, which confirms that under certain conditions, the NTLI can reflect the housing price of a district, and there is a significant positive correlation between them. The R^2^ and R^2^ adjusted values of the OLS are less than 0.28. The AIC_c_ value of the GWR is smaller than that of the OLS, which means that the GWR is more suitable for regression fitting of the NTLI and CHP. Comparing the GWR and OLS results, we find that the GWR model considering the influence of geographical location on equation fitting is better than the OLS model in fitting the experimental data.

Through an interactive validity test, the optimal bandwidth of the GWR model is 0.114. The experimental results show that the NTLI in Wuhan has obvious spatial non-stationarity through the GWR to predict the housing price.

### 3.3. Discussion

The coupling mechanism between the NTLI and CHP is analyzed from two aspects as follows:

(1) From the perspective of the spatial clustering analysis:

The NTLI in Wuhan shows a pattern of spatial agglomeration, and the NTLI decreases from the city center to the suburbs. That is, the activities of people in the city center are more concentrated, and the night lighting facilities in the city center are more widespread and perfect than those in the suburbs. Overall, the NTLI shows a downward trend from the central area to the peripheral area. The CHP in Wuhan decreases from the city center along the traffic ring to the suburbs, forming a circle-like spatial pattern. That is, the closer a city is to the city center, the higher the level of living consumption. In addition, in the outer edge of the city, housing prices tend to show a downward trend from the center to the outside. Therefore, there is a strong linear positive correlation between the CHP and NTLI in Wuhan.

There are some outliers both in the CHP and the NTLI, mostly between the HH cluster and LL Cluster. This is because there is a wide gap between the rich and poor China. High-price communities tend to be concentrated in the city center, while suburban housing prices are generally lower. In the transitional zone between the urban centers and suburbs, the communities with high prices and those with low prices are interlaced.

(2) From the perspective of the spatial quantitative analysis:
  (a) Comparing the results of the GWR with the OLS estimation, it is found that the fitting effect of the GWR estimation is better than that of the traditional OLS estimation. This is because the OLS estimation does not consider the factors of spatial distance, and the results only describe the effect of night-time light on housing prices in general. It is a global estimation that cannot reflect the heterogeneity of parameters in space. The GWR model reveals the information that cannot be explained by the OLS to reflect changes in the CHP.
  (b) There is a significant spatial heterogeneity in the NTLI in Wuhan, which is reflected in the obvious spatial difference between the NTLI and CHP.
  (c) The GWR model provides technical support for the quantitative measurement of structural changes in spatial variables. It can measure the variation in influencing factors in local geographic space. It has a prospect of wide application in studying the non-stationarity of the influencing factors of housing prices.

## 4. Conclusions

This paper uses Wuhan as the research area and CHP data as the research object, combined with Luojia1-01 night-time light imagery, and carried out small-scale spatial correlation analysis and modeling. The experimental results show that there is a strong linear positive correlation between the NTLI and CHP, the CHP data in Wuhan has obvious spatial non-stationarity, and there is potentiality of estimating the CHP by using Luojia1-01 night-time light imagery

Through the spatial analysis of the CHP and NTLI in Wuhan, we can find the intrinsic relationship between them and simultaneously fill the gap in the research field of small-scale night-time light to a certain extent. The experimental results show that this study has certain theoretical and practical significance.

In terms of theoretical significance, since the launch of the Luojia1-01 satellite in June 2018, many scholars have made use of the image data of the Luojia1-01 satellite to carry out relevant research, but generally speaking, there is too little research on the Luojia1-01 data. This paper is the first to use the Luojia1-01 night-time light data to carry out small-scale housing price research, which is an extension of the application of the Luojia1-01 data.

In terms of practical significance, this study contributes to revealing the coupling mechanism between the CHP and NTLI. The ability to predict and analyze the impact of the NTLI on CHP is not only an important issue of concern to residents, developers, and government departments but also plays an important role in real estate market policy-making and urbanization development. Through the prediction of the CHP and the analysis of the influence of the NTLI in Wuhan, this paper can provide guidance for consumers to purchase housing to a certain extent, provide some reference for developers and investors, provide reference for relevant government departments, help strengthen the management of real estate, and contribute to the development of urbanization.

However, there are still some shortcomings in this study, and there are still some aspects that need to be improved.

(1) As a new generation of night-time light images, Luojia1-01 provides higher spatial resolution, a wider radiation measurement range, and richer urban dynamic information than previous night-time light data. However, the lack of multi-temporal images limits its application in time series. By integrating Luojia1-01 with other high-resolution night-time light images, we can find a solution to this problem. Considering spatial non-stationarity and temporal non-stationarity will be one of the next research priorities of this method.
(2) The experimental results show that there is a strong linear positive correlation between the NTLI and CHP. How to quantitatively evaluate the explanatory power of the NTLI to CHP is also a problem to be discussed in this paper.

## Figures and Tables

**Table 1:** *Global Moran’s I* summary.

Variable	CHP	NTLI
Moran’s I index	0.254795	0.244385
Expectation index	−0.000426	−0.00426
Variance	0.000001	0.000001
Z score	208.965341	200.756958
*p* value *	0.000000	0.000000

* In ArcGIS, the *p* value can only be given to six decimal places. When the *p* value is too small, it is approximated to zero.

**Table 2:** *General G* summary.

Variable	CHP	NTLI
Observation value of *General G*	0.208858	0.271641
Expected value of *General G*	0.169386	0.169386
Variance	0.000002	0.000001
Z score	28.090046	32.110155
*p* value	0.000000	0.000000

**Table 3:** Results of Geographically weighted regression (GWR) and Ordinary least square (OLS).

Variable	GWR Value	OLS Value
Bandwidth	0.114996	-
ResidualSquares	2.07E+10	-
Sigma	4230.55	-
AICC	22738.6	23098.122853
R^2^	0.518962	0.281875
R^2^ Adjusted	0.516875	0.280009
Trace_of_Smatrix	52.6018	-
F-Stat	-	150.988220
Wald	-	243.623832
Wald-Prob	-	0
K(BP)	-	28.721512
K(BP)-Prob	-	0
JB	-	1983.028524
JB-Prob	-	0
Sigma2	-	26788093.986300
